# Isometric scaling to model water transport in conifer tree rings across time and environments

**DOI:** 10.1093/jxb/eraa595

**Published:** 2020-12-24

**Authors:** Irina V Sviderskaya, Eugene A Vaganov, Marina V Fonti, Patrick Fonti

**Affiliations:** 1 Siberian Federal University, Krasnoyarsk, Russian Federation; 2 V.N. Sukachev Institute of Forest, Siberian Branch of the Russian Academy of Sciences, Krasnoyarsk, Russian Federation; 3 Dendrosciences, Swiss Federal Institute for Forest, Snow and Landscape Research WSL, Zürcherstrasse, Birmensdorf, Switzerland; 4 Hong Kong Baptist University, Hong Kong

**Keywords:** Bordered pits, conifer, hydraulic properties, Pinaceae, tracheid, tree ring, xylem

## Abstract

The hydraulic properties of xylem determine the ability of plants to efficiently and safely provide water to their leaves. These properties are key to understanding plant responses to environmental conditions and evaluating their fate under a rapidly changing climate. However, their assessment is hindered by the challenges of quantifying basic hydraulic components such as bordered pits and tracheids. Here, we use isometric scaling between tracheids and pit morphology to merge partial hydraulic models of the tracheid component and to upscale these properties to the tree-ring level in conifers. Our new model output is first cross-validated with the literature and then applied to cell anatomical measurements from *Larix sibirica* tree rings formed under harsh conditions in southern Siberia to quantify the intra- and inter-annual variability in hydraulic properties. The model provides a means of assessing how different-sized tracheid components contribute to the hydraulic properties of the ring. Upscaled results indicate that natural inter- and intra-ring anatomical variations have a substantial impact on the tree’s hydraulic properties. Our model facilitates the assessment of important xylem functional attributes because it requires only the more accessible measures of cross-sectional tracheid size. This approach, if applied to dated tree rings, provides a novel way to investigate xylem structure–function relationships across time and environmental conditions.

## Teaser

A model uses isometric scaling within conduit elements to facilitate the assessment of wood hydraulic properties. This allows the use of tree rings to reconstruct these characteristics across time and environments.

## Introduction

The xylem of plants provides an important hydraulic pathway for sap to reach the leaves, where photosynthesis occurs (Tyree and Zimmermann, 2002; Holbrook, 2005). The ability of a plant to survive and perform therefore depends on how well the functional properties of this pathway are adapted to local environmental conditions. If this pathway does not facilitate enough transport capacity during optimal conditions (usually quantified as hydraulic conductivity), or if it fails to function during unfavorable periods (quantifiable as vulnerability to embolism), the plant will eventually become maladapted and die (Venturas *et al.*, 2017). The capacity to cope with climate change, such as the increased risk of drought (e.g. Brodribb and Cochard, 2009) and frost (e.g. Pockman and Sperry, 1996), will therefore hinge on the ability of the plant to build an adequate and functional xylem structure despite the numerous environmental and ontogenetic constraints. Understanding the relationships between the environment, wood structure, and its functioning is therefore fundamental to predicting the fitness, function, survival, and distribution of plants at a global scale (e.g. McDowell, 2011). Unfortunately, assessing these relationships across time and space remains a significant challenge.

Assessing the functional properties of xylem in mature trees is not always an easy task (Boyer, 1995; Melcher *et al.*, 2012). Although measurements of hydraulic conductivity and vulnerability to embolisms are essential for quantifying the functional status of a plant’s hydraulic system, these assessments are often invasive or limited to young branches. To overcome this issue, some studies have attempted to link structure and function via the calibration of relatively rapid and integrative assessments of wood technological properties. These include wood density (Dalla-Salda *et al.*, 2011), ultrasonic waves (De Roo *et al.*, 2016), and Fourier transform infrared spectroscopy (Tsuchikawa and Kobori, 2015; Savi *et al.*, 2019). However, all of these approaches fail to include variable wood cell structure in their analysis, as it is very difficult to link hydraulic characteristics to specific wood structural elements, such as annual rings or single conduits (but see e.g. Zwieniecki *et al.*, 2001; Mayr and Cochard, 2003; Christman and Sperry, 2010; Nolf *et al.*, 2017). However, hydraulic or computational models of pit and tracheid hydraulic properties (e.g. Lancashire and Ennos, 2002; Valli *et al.*, 2002; Hacke *et al.*, 2004) are an effective alternative for directly linking structural elements to their function. These models have also been combined using average conduit characteristics to scale up to the tissue level (Wilson *et al.*, 2008; Tanrattana *et al.*, 2019). Yet, there are no models that relate hydraulic properties and performance at the wood tissue level considering the anatomical variability of conduit elements.

However, scaling up hydraulic models to the tissue scale would eventually allow for the consideration of sapwood as a heterogeneous and variable tissue composed of differently sized tracheids. This would result in more accurate biological representations of the relationship between wood structure and hydraulic performance, and of how these relationships are influenced by the environment. To broadly investigate the impact of global change on plant hydraulics, it is therefore necessary to account for the natural variability of wood tissue at the subcellular scale, for example, via the quantification of isometric scaling among the hydraulic structural elements of xylem.

Scaling relationships between body components of living organisms are very common in nature (Gould, 1966). These relationships have also been confirmed to govern plant vascular systems (West *et al.*, 1999). Tapering of water-conducting cells with tree size has been quantified and is quite stable across species and environments (Anfodillo *et al.*, 2006; Olson *et al.*, 2014; Williams *et al.*, 2019; Soriano *et al.*, 2020). The same tip-to-base conduit widening has also been observed along the stem radius, from pith to bark (Carrer *et al.*, 2015). Recently, similar relationships have been observed for tracheids and pit sizes measured along the stem axis of a giant sequoia (Lazzarin *et al.*, 2016). This scaling might serve to prevent any single component from generating a disproportionate amount of the total conduit resistance and maintain sufficient efficiency of the tree hydraulic system (Choat *et al.*, 2008; Williams *et al.*, 2019). Direct empirical evidence for such proportionality has been provided among species (Wheeler *et al.*, 2005; Hacke *et al.*, 2006; Pittermann *et al.*, 2006) and is presumed also to apply intraspecifically (Domec *et al.*, 2008; Lazzarin *et al.*, 2016; Jacobsen *et al.*, 2018). The nature of these scaling relationships can be associated with common and coordinated developmental processes. For example, the mechanism of pit formation described by Savidge (2014) can explain the stable size scaling between tracheids and pits. A tracheid spending more time in the enlarging phase has more time to increase in size (see Anfodillo *et al.*, 2012) and to generate proportionally larger nascent bordered-pit organelles (i.e. roughly spherical objects associated with bordered-pit formation; see Savidge 2014). It is thus legitimate to assume that scaling occurs at the tracheid level, that is, between the size of the tracheid and the structural characteristics of its pits and membranes.

This presumed proportionality could greatly facilitate assessments of structure–function responses of xylem to environmental variability because it would be necessary to measure only the cross-sectional tracheid lumen diameter and cell wall thickness. These measurements have improved enormously thanks to significant advances in wood sectioning (e.g. Gartner *et al.*, 2015) and image analysis-supported anatomical measurements (e.g. von Arx and Carrer, 2014). Moreover, analytical tools such as the R package *RAPTOR* (Peters *et al.*, 2018) enable the integration of tree-ring measurements into a representative radial profile (i.e. a tracheidogram; see (Теrskov *et al*., 1981; Vysotskaya and Vaganov, 1989; Vaganov *et al.*, 2006). Thus, the stage is set for a tree-ring-based investigation of environment–structure–function relationships of conifer species across time and space.

**Fig. 1.**
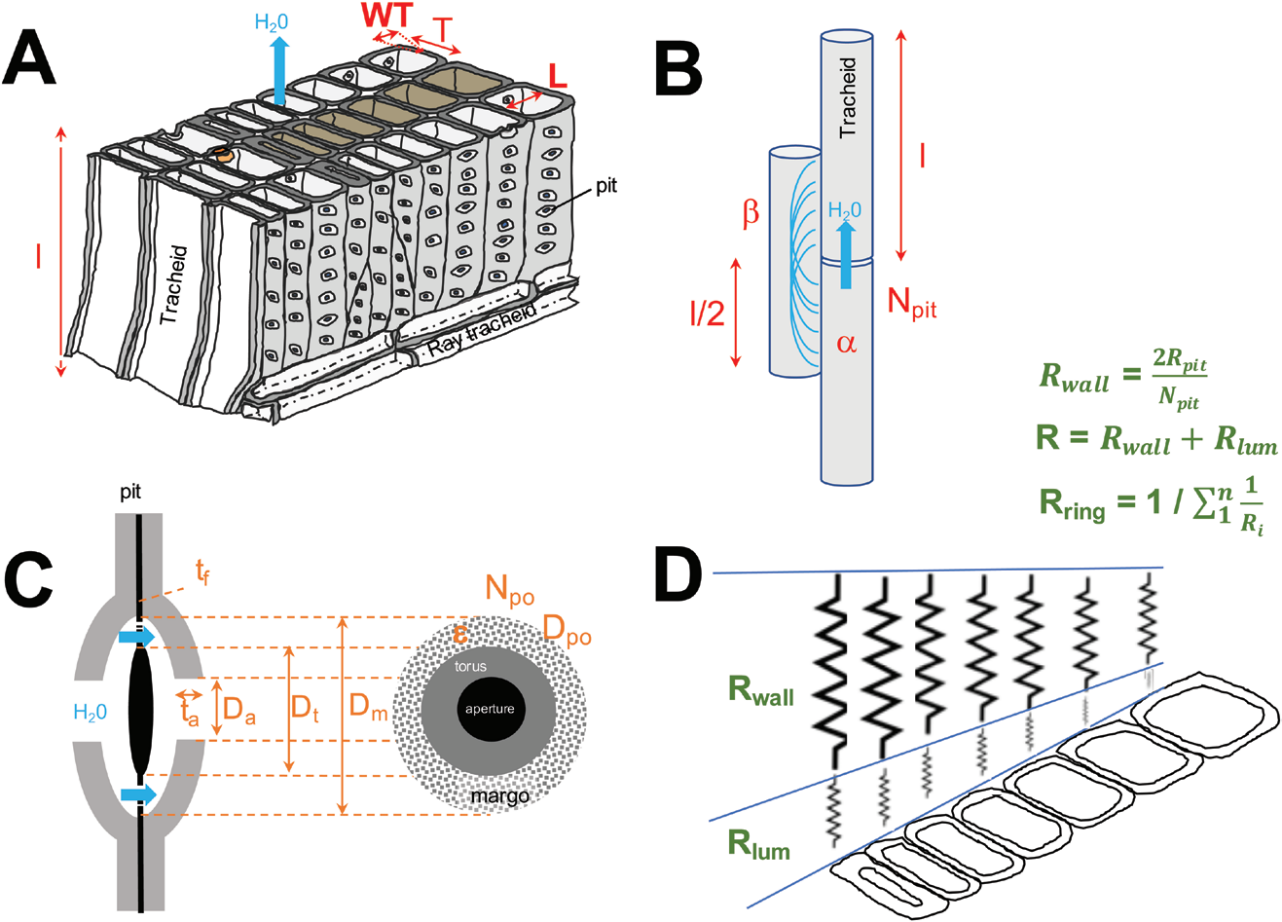
Schema of the hydraulic model. (A) Simplified three-dimensional schema representing the structure of conifer wood. The model assesses the hydraulic properties (conductance and resistance) of the water flowing up the stem via the lumen and the walls of networking tracheids of length *l*, radial and tangential lumen diameter *L* and *T*, and wall thickness *WT*. Shaded cells indicate a radial file of tracheids along an annual ring. (B) Schema showing the path of water between neighboring tracheids. Each molecule of water travels up an average distance of half the tracheid length (β) before entering the next tracheid via bordered pits, the number of which (*N*_pit_) is defined by the pit density α. (C) Transverse and radial views of a bordered pit between two neighboring tracheids. Water flows from one tracheid to the next via the bordered pit aperture and through a porous membrane characterized by a number of pores (*N*_po_) of average diameter (*D*_po_). *D*_a_, *D*_m_, and *D*_t_ indicate the diameter of the aperture, the torus, and the margo, respectively. *t*_a_ and *t*_f_ characterize the channel depth and the thickness of the margo, respectively, and ε indicates the fraction of margo area occupied by pores. (D) Integration of the tracheid hydraulic properties at the scale of a radial file. The total radial file resistance (*R*_ring_) corresponds to the sum of the resistance (*R*) of each tracheid in a series. *R* is calculated as the sum of the tracheid lumen (*R*_lum_) and wall (*R*_wall_) resistances. The tracheid wall resistance corresponds to the integration of all the pit resistances (*R*_pit_) in parallel. Abbreviations identify the variables quantified by the model (see Table 1 for definitions). Labels in red text refer to variables at the tracheid scale, those in orange to bordered pit-scale variables, and those in green to variables at the tree-ring scale.

Here, based on a literature evaluation of isometric size relationships between a tracheid and the morphology and number of its bordered pits, we propose, apply, and discuss a novel model allowing an evaluation of the hydraulic properties of conifer tree rings. Specifically, our model combines existing partial models to quantify the hydraulic conductance and resistance of single pits and tracheids. As the only inputs, we use tracheid size (lumen and wall cross-section) data. This will allow us to integrate the results at the tissue level, for example, for a given tree ring (Fig. 1A). In particular, we hypothesize that the use of isometric relationships will allow quantification of the contributions of the pits and tracheid lumen to the total ring resistance and their variation across tree rings formed under differing environmental conditions. As a result, our model is suitable for long-term, high-resolution plant ecological studies.

## Materials and methods

### The hydraulic model

The conifer tree-ring hydraulic model we propose is aligned with the generally accepted cohesion-tension theory of transpiration-pulled sap flowing through a network of variously sized tracheid lumina connected via bordered pits (Tyree and Zimmermann, 2002). The model comprises existing independent partial models of pit (Hacke *et al.*, 2004; Pittermann *et al.*, 2006) and tracheid hydraulics (Lancashire and Ennos, 2002; Wilson *et al.*, 2008), and innovatively combines them by postulating isometric size relationships between bordered pits and tracheid structures. This allows the modeling of water transport at the tree-ring scale by using only tracheid anatomical measures (Fig. 1). Scaling up of the model is made possible by the fact that almost all the conifer sapwood area is devoted to conduction and that more than 90% of xylem cells are water-conducting tracheids. The model alleviates the need for pit measurements, which are difficult to obtain. The overall tree-ring hydraulic properties are thus modeled as functions of tracheid size (e.g. their length, lumen, and wall thickness) (Fig. 1A), their overlap (Fig 1B), and the density and dimensions of the bordered pits and pores in the margo (Fig. 1C). The constants, parameters, and variables used in the model are described and summarized in Table 1. The hydraulic model quantifies two hydraulic properties, the hydraulic conductance (*K*) and the corresponding hydraulic resistance (*R*). These hydraulic properties are obtained by integrating these properties at each structural level, from the single pit up to the tree ring. Scaling down from the tree ring to the pit, each level of the model is described as follows.

**Table 1.** Model constants, parameters, and variables

Abbreviation	Description	Equation	Integration scale	References
	**Constant**			
μ	Dynamic viscosity of water at 20 °C = 1.002×10^–9^ kg s^–1^ mm^–1^			
	**Parameters**			
*t*_f_	Mean thickness of margo strands = 140 nm		Margo	(Domec *et al.*, 2006)
*l*	Axial tracheid length = 2.2 mm		Tracheid	(Arseneva and Chavchavadze, 2001)
*TD*	Tracheid tangential diameter = 30 µm		Tracheid	
α	Pit density = 6.5×10^8^ m^–2^		Tracheid	(Bailey and Tupper, 1918; Thomas and Scheld, 1967; Lin, 1989; Boyer, 1995; Domec *et al.*, 2006)
β	Factor of tracheid overlap = 0.5		Tree-ring	(Kedrov, 2012)
	**Input variables**			
*L*	Radial lumen diameter		Tracheid	
*WT*	Wall thickness		Tracheid	
*i*	Tracheid position in the tracheidogram		Tree-ring	
	**Calculated variables**			
*T*	Tangential lumen diameter = 30 µm–2WT		Tracheid	
*D*_h_	Tracheid hydraulic diameter	4	Tracheid	
*N*_pit_^*a*^	Number of pits per tracheid	7		
*D*_po_	Mean diameter of the pores in the margo		Margo	
*N*_po_	Number of pores in the margo		Margo	
*D*_pe_^*a*^	Equivalent diameter of margo pores	10	Margo	
*D*_m_^*a*^	Pit membrane diameter		Pit	
max*D*_m_^*a*^	Maximum pit membrane diameter		Pit	
*D*_t_^*a*^	Torus diameter		Pit	
*D*_a_^*a*^	Diameter of pit aperture		Pit	
*D*_mc_	Diameter of the circle whose area equals the difference between membrane and torus		Pit	
*t*_a_	Pit canal length (equals WT)		Pit	
ɛ	Fraction of margo area occupied by pores		Pit	(Tio and Sadhal, 1994; Hacke *et al*., 2004)
	**Model output**			
*R*, *K*	Tracheid resistance and conductance	3, 8	Tracheid	
*R*_lum_, *K*_lum_	Lumen resistance and conductance	5	Tracheid	
*R*_wall_, *K*_wall_	Wall resistance and conductance	6, 9	Tracheid	
*R*_pit_	Individual pit resistance	9	Pit	
*R*_canals_, *R*_apertures_, *R*_margo_	Resistance of the pit canals, apertures, and margo	9	Pit	
*R*_ring_, *K*_ring_	Radial file resistance and conductance	1, 2	Radial file (tree-ring)	
*Pit*_contr_	Contribution of pits to the total ring resistance	11, 12	Radial file (tree-ring)	
*R*_onlylum_	Ring resistance consisting of parallel lumen only	12	Radial file (tree-ring)	

**Fig. 2.**
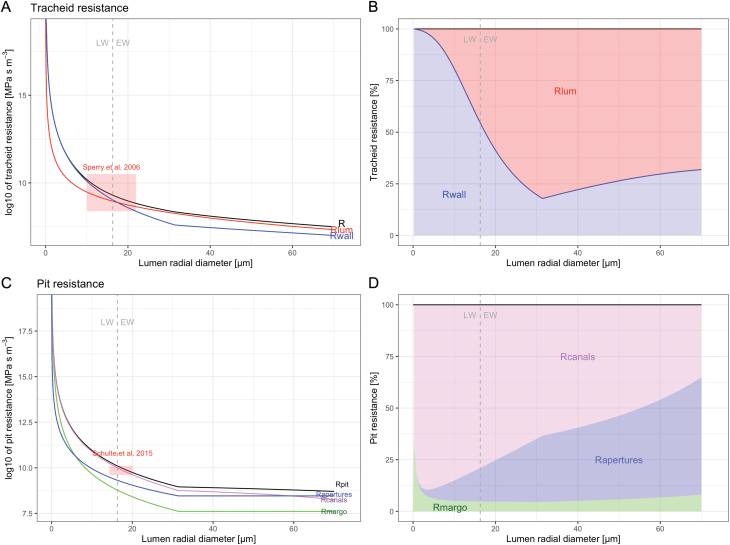
Modeled resistances for a tracheid (A, B) and an individual pit (C, D) with increasing tracheid size. Isometric relationships between tracheid size and pit size applied in the model are indicated in Table 2. To facilitate the representation of model output, model input parameters have been fixed as follows: tracheid length *l* = 2.2 mm, tracheid tangential lumen diameter *TD* = 30 μm, maximum pit size max*D*_m_ = 22 μm, pit density α = 6.5×10^8^ m^–2^, mean thickness of margo strands *t*_f_ = 140 nm, and pith canal length *t*_a_ = tracheid wall thickness (*WT*). The *WT* is set to increase constantly according to *WT* = –8/70 × *L* + 10. A and C show the absolute values on a logarithmic scale (log_10_); B displays the contributions of the two hydraulic components (blue, tracheid walls; red, tracheid lumen) of the overall tracheid resistance; and D displays the contributions of the pit margo (green), pit apertures (blue) and pit canals (violet) of the total pit resistance. The dotted grey vertical line defines the transition between an earlywood (EW) and latewood tracheid according to the Mork’s index definition, where EW = *L* > 2 × *WT* (Denne, 1988). The red rectangles in A and C show the corresponding range of resistances as observed in Schulte *et al.* (2015) and (Sperry *et al.* (2006). To allow comparison, the data in Sperry *et al.* (2006) have been transformed from conductivity to resistance.

Model parameters have been fixed to facilitate the representation of tracheid size on model output (see Fig. 2). However, these can be made variable and adjusted according to specific characteristics of the species considered.

^*a*^ Estimated using isometric relationships (see Table 2).

#### Tree ring

For the sake of simplicity, we illustrate the tree ring as a representative radial file of tracheids, that is, a profile of tracheid radial size and wall thickness across tree rings as represented by a tracheidogram (see Vaganov *et al.*, 2006). The tree-ring hydraulic properties therefore correspond to the integration of the hydraulic properties of the tracheidogram. Since a tracheid mainly exchanges water with its tangential neighbors, we consider the representative radial file as a set of isolated parallel hydraulic resistances (Fig. 1D; see also Zimmermann *et al.*, 1971; Calkin *et al.*, 1985; Calkin *et al.*, 1986; Schulte *et al.*, 1987; Schulte and Gibson, 1988; Kedrov, 2012). Thus, the total ring conductance (*K*_ring_) equals the sum of the hydraulic conductance (*K*_i_) of all tracheids:

$$\begin{array}{l} K_{ring}=\underset{i=1}{\overset{n}{\sum }}\;K_{i} \end{array}$$ (1)

Because the resistance is the inverse of the conductance, the resistance *R*_ring_ is:

$$\begin{array}{l} R_{ring}=\;\;\;\frac{1}{K_{ring}}=\;\;\;\frac{1}{\overset{n}{\underset{i=1}{\sum }}\frac{1}{\;\;\;R_{i}}} \end{array}$$ (2)

where *K*_i_ and *R*_i_ are the conductance and resistance of each tracheid in the radial file, respectively.

#### Tracheid

Since water flows from one tracheid to the next via the bordered pits incorporated in the cell wall, the total flow resistance of a single tracheid (*R*) corresponds to the summed lumen (*R*_lum_) and wall/pit resistances (*R*_wall_):

$$\begin{array}{l} R=R_{lum}+R_{wall} \end{array}$$ (3)

The lumen resistance *R*_lum_ is calculated according to the Hagen–Poiseuille equation considering a hydraulic diameter *D*_h_ from the rectangular lumen cross-section as:

$$\begin{array}{l} D_{h}=\frac{2L\times T}{L+T} \end{array}$$ (4)

where *L* and *T* are the radial and tangential lumen diameters, respectively (Lewis, 1992; White, 1991). The lumen resistance is thus calculated as:

$$\begin{array}{l} R_{lum}=\frac{8\mu \beta l{\left(L+T\right)}^{4}}{\pi L^{4}\;T^{4}} \end{array}$$ (5)

where µ is the dynamic viscosity of water at 20 °C, *l* is the tracheid length, and *β*=0.5 is the proportion of tracheid overlap, that is, considering that water travels only half the length of the tracheid before crossing through a pit into the next cell (see Fig. 1B; Lancashire and Ennos, 2002).

The resistance of the wall *R*_wall_ is equivalent to the resistances of all the pits in parallel. The model also assumes that all the pits of a tracheid are of the same size and are evenly distributed between the two radial walls. Consequently, the total wall resistance *R*_wall_ corresponds to twice the tracheid wall resistance (i.e. one for each side of the tracheid):

$$\begin{array}{l} R_{wall}=2\times \frac{1}{\overset{n}{\underset{i=1}{\sum }}\frac{1}{R_{pit}}}\;=\;2\;\frac{R_{pit}}{N_{pit}} \end{array}$$ (6)

where *R*_pit_ is the resistance of each pit on the wall and *N*_pit_ is the total number of pits. The number of pits is the product of the tracheid radial wall area (tracheid length *l* × lumen radial diameter *L*) and the pit density (*α*), that is, the number of pits per unit of radial wall area:

$$\begin{array}{l} N_{pits}=\alpha \times L\times l \end{array}$$ (7)

where *α* is 6.5×10^9^ m^–2^. So, the extended equation for the tracheid resistance is:

$$\begin{array}{l} R=R_{lum}+R_{wall}=\frac{8\mu \beta l\times {\left(L+T\right)}^{4}}{\pi L^{4}T^{4}}+\frac{2R_{pit}}{\alpha Ll} \end{array}$$ (8)

#### Pit

The individual pit resistance *R*_pit_ is determined following Hacke *et al.* (2004), who sum the partial resistances of the margo (*R*_margo_) with those of the pit canals (*R*_canals_) and pit apertures (*R*_apertures_). The partial resistances are computed as a function of the number of pores in the margo (*N*_po_), the equivalent pore diameters of the margo (*D*_pe_), the pit aperture diameter (*D*_a_), and the canal length (*t*_a_), according to the following equation:

$$\begin{array}{ll} R_{pit}= & \;R_{margo}+2R_{canals}+2R_{apertures}=\frac{24\mu }{n_{po}D_{pe}^{3}}f\left(\varepsilon \right)\\ & +2\frac{128\cdot t_{a}\cdot \mu }{\pi D_{a}^{4}}+2\frac{24\mu }{D_{a}^{3}} \end{array}$$ (9)

where µ is the dynamic viscosity of water at 20 °C, ɛ is the fraction of margo area occupied by equally sized pores, and f(ε) is the correction function considering the interaction of streams of water moving through neighboring pores in the margo (Tio and Sadhal, 1994). The number of pores in the margo (*N*_po_) is calculated following Hacke *et al.* (2004) as:

$$\begin{array}{l} N_{po}=\;\;\;\frac{D_{m}^{2}}{{\left(0,63D_{p}+t_{f}\right)}^{2}} \end{array}$$ (10)

where *t*_f_ is the mean thickness of strands in the margo and *D*_mc_ is the diameter of a circle whose area equals the difference between the total membrane area and the torus area.

The contribution of the pits to the total ring resistance (*Pit*_contr_) is calculated as:

$$\begin{array}{l} Pit_{contr}=1-\frac{R_{onlylum}}{R_{ring}} \end{array}$$ (11)

where *R*_ring_ is the total ring resistance (or the inverse of the total ring conductance; see equation 2) and *R*_onlylum_ is the resistance of the ring consisting of parallel lumens only. *R*_onlylum_ is calculated as:

$$\begin{array}{l} R_{onlylum}={\left(\underset{i=1}{\overset{N}{\sum }}\;\frac{1}{R_{onlylum_{i}}}\right)}^{-1} \end{array}$$ (12)

where *R*_*lum*_ is the lumen resistance of each tracheid in the tracheidogram.

### Input data and model run

The isometric scaling between tracheids and pits applied in this study was assessed by exploring the wood hydraulic and anatomical bibliography of Pinaceae and cross-validated by comparing partial model outputs to related results from independent studies. We explicitly selected this family because the diameter of the pit aperture varies with the diameter of the tracheid (Carlquist, 1988). To assess the influence of the isometric relationship at both the pit and the tracheid level, the corresponding hydraulic properties are calculated for each level of integration, from pit to tree ring.

To scale up results at the tree-ring level, the model was run using tracheid and pit anatomical data from a *Larix sibirica* stand (at 54.2517 N, 89.6136 E, 550 m a.s.l.) located near Shira in southern Siberia. The climate there is continental cold (annual average temperature 0.8 °С) and dry (total annual precipitation 294 mm). The stand is composed of mature trees ~19 m tall with stem diameters at breast height of ~30 cm. Cross-sectional tracheid anatomical features [*L* and wall thickness (*WT*)] were measured from magnified images of 12 μm thick micro-sections of wood cores taken at stem heights of 1.3 m. Measurements focused on rings produced over the period from 1986 to 2015. The micro-sections were cut with a sliding microtome (HM 450, Thermo Scientific, USA) and stained with Safranin and Astrablue before being fixed permanently into Euparal (Gartner *et al.*, 2015). Tracheid measurements were performed on magnified images (2.361 pixels per μm) captured with a digital camera (Canon EOS 650D, Canon Inc., Tokyo, Japan) connected to an Olympus BX41 light microscope (Olympus Corp., Tokyo, Japan) using *ROXAS* (von Arx and Carrer, 2014). Radial files were recognized with the R package *RAPTOR* (Peters *et al.*, 2018) and the 10 tangentially largest files per ring were averaged to obtain a representative radial file (tracheidogram) for each ring using the R package *TracheidR* (Campelo *et al.*, 2016). The R programming environment (CRAN: http://cran.r-project.org) was used to formulate and run the model. Although tracheid length can vary slightly within the ring (Fabisiak *et al.*, 2020; Arseneva and Chavchavadze, 2001), the tracheid length for our calculations was set to 2.2 mm. In addition, pit measurements were taken to relate pit diameter (*D*_m_) to tracheid lumen diameter (*L*). These measurements were taken in order to cover the full range of tracheid size, and were obtained by measuring 100 selected pits on 12 μm thick radial cross-sections. Measurements were performed on digital images collected with a slide scanner (Axio Scan.Z1, Zeiss, Germany) at a resolution of 2.265 pixels per μm.

## Results

### Isometric relationships and cross-validation

The bibliographic search revealed several studies quantifying linear relationships among and within bordered pits and tracheids in the Pinaceae family (Table 2). Linear relationships have been observed between the tracheid size (lumen radial diameter) and the pit number (*N*_pit_) and size (i.e. the diameter of the pit membrane, *D*_m_). Anatomical observations indicate a limitation on pit size even in wider tracheids (e.g. Lazzarin *et al.*, 2016). This usually happens in wider tracheids displaying two longitudinal rows of pits (e.g. Takizawa, 1974). In our model, we control for this isometric relationship by setting a maximum pit size (max*D*_m_), despite the existence of larger tracheids. This leveling off occurs according to the following equation:

**Table 2.** Isometric relationships for the Pinaceae family as observed in the literature

Isometric relationships	Range of observations from the references	Species	References
*D*_m_= 0.70 *L* (±0.07) up to a max*D*_m_, then fixed to max*D*_m_	*L* 6–35 µm	17 gymnosperm species	(Hacke *et al.*, 2004)
*D*_t_ = 0.50 *D*_m_ (±0.11) *D*_a_ = 0.25 *D*_m_ (± 0.04)	*D*_m_ 8–24 µm	*Pinus cembra*	(Domec *et al.*, 2006; Yaman, 2007; Hacke and Jansen, 2009; Schulte, 2012; Schulte *et al.*, 2015; Losso *et al.*, 2018)
		*Picea abies*	
	*D*_a_ 2–7 µm	*Picea glauca*	
		*Abies balsamea*	
	*D*_t_ 5–12 µm	*Larix laricina*	
		*Pseudotsuga menziesii*	
		*Picea mariana*	
*D*_po_ = 0.03030 *D*_m_ (±0.0025)	*D*_po_ 0.03–0.07 µm	*Pseudotsuga menziesii*	(Domec *et al.*, 2006)
*t*_a_= *WT*	µm		

*L*, tracheid lumen diameter; *D*_m_, diameter of pit membrane; max*D*_m_, maximum membrane diameter; *D*_t_, diameter of pit torus; *D*_a_, diameter of pit aperture; *D*_po_, diameter of pores in the margo; *t*_a_, pit canal length; *WT*, tracheid wall thickness.

$$\begin{array}{l} f\left(D_{m}\right)=\left\{ \begin{array}{l} D_{m}\;=0.7\times L,\;D_{m}<maxD_{m}\\ D_{m}\;=maxD_{m},\;D_{m}\geq maxD_{m} \end{array}\right. \end{array}$$ (13)

Similarly, linear relationships have been assessed between the diameter of the pit membrane (*D*_m_) and the diameters of the torus (*D*_t_), the pit aperture (*D*_a_), and the pores in the margo (*D*_po_). Although pore sizes in a margo can vary considerably and are unrelated to the membrane diameter (Domec *et al.*, 2006), there are indications of a negative relationship between air-seed pressure and the margo diameter (Hacke *et al.*, 2004). This led us to simplify the model with the assumption that pores in a margo are of the same size and are related to the membrane diameter.

These associations, combined with model parameters similarly extracted from the literature (e.g. for the mean thickness of margo strands *t*_f_, the pit density α (Domec *et al.*, 2006), and the pith canal length *t*_a_—that is, the tracheid double wall thickness), allowed us to model both pit and tracheid hydraulic components as a function of tracheid size (Fig. 2). As expected, both the pit and lumen resistances decrease exponentially with increasing lumen diameter. The total resistance for a 10 μm-wide radial lumen tracheid is 15.72×10^9^ MPa s m^–3^, with a contribution of pits of 80% (12.68×10^9^ MPa s m^–3^). These values are reduced by two orders of magnitude to 94.60×10^*7*^ MPa s m^–3^ and 39.01×10^*7*^ MPa s m^–3^ (with a contribution of pit resistance of 41%), respectively, if the radial lumen is twice the size (i.e. 20 μm). Considering the Mork index (Denne, 1988) as the criteria to separate earlywood and latewood, the reduction in the latewood is much stronger than in the earlywood. Notably, the predominant contribution to the total tracheid resistance in the latewood is determined by the pits, but switches to the lumen in the earlywood (Fig. 2B). The conductance of pits in the earlywood tracheids is limited by the torus, whereas the conductance of pits in the latewood tracheids is limited by pit canal length. Margo resistance (*R*_margo_) plays only a minimal role in determining the total pit resistance compared with the contribution of the pit canal resistance (*R*_canals_, dominant in the latewood) and the aperture resistance (*R*_apertures_), which become increasingly relevant with increasing tracheid lumen diameter (Fig. 2D). Since in earlywood tracheids the pit canal can be very thin (see e.g. Sano and Nakada 1998), *R*_canals_ of earlywood tracheids might be negligible.

As a benchmark to cross-validate our model output, we identified two experimental studies assessing the hydraulic properties of single pits (Schulte *et al.*, 2015) and tracheids (Sperry *et al.*, 2006). For comparison, we calculated the resistances for the same pit metrics as those studied by Schulte *et al.* (2015) in *Picea mariana* (black spruce) and *Picea glauca* (white spruce) (indicated by the red rectangle in Fig. 2C). Similar comparisons have also been performed for the hydraulic properties of single conifer tracheids as assessed by Sperry *et al.* (2006) (indicated by the red rectangle in Fig. 2A). In both cases, the ranges of these data overlap well with our modeled resistances at comparable pit and tracheid sizes.

### Model output

**Fig 3.**
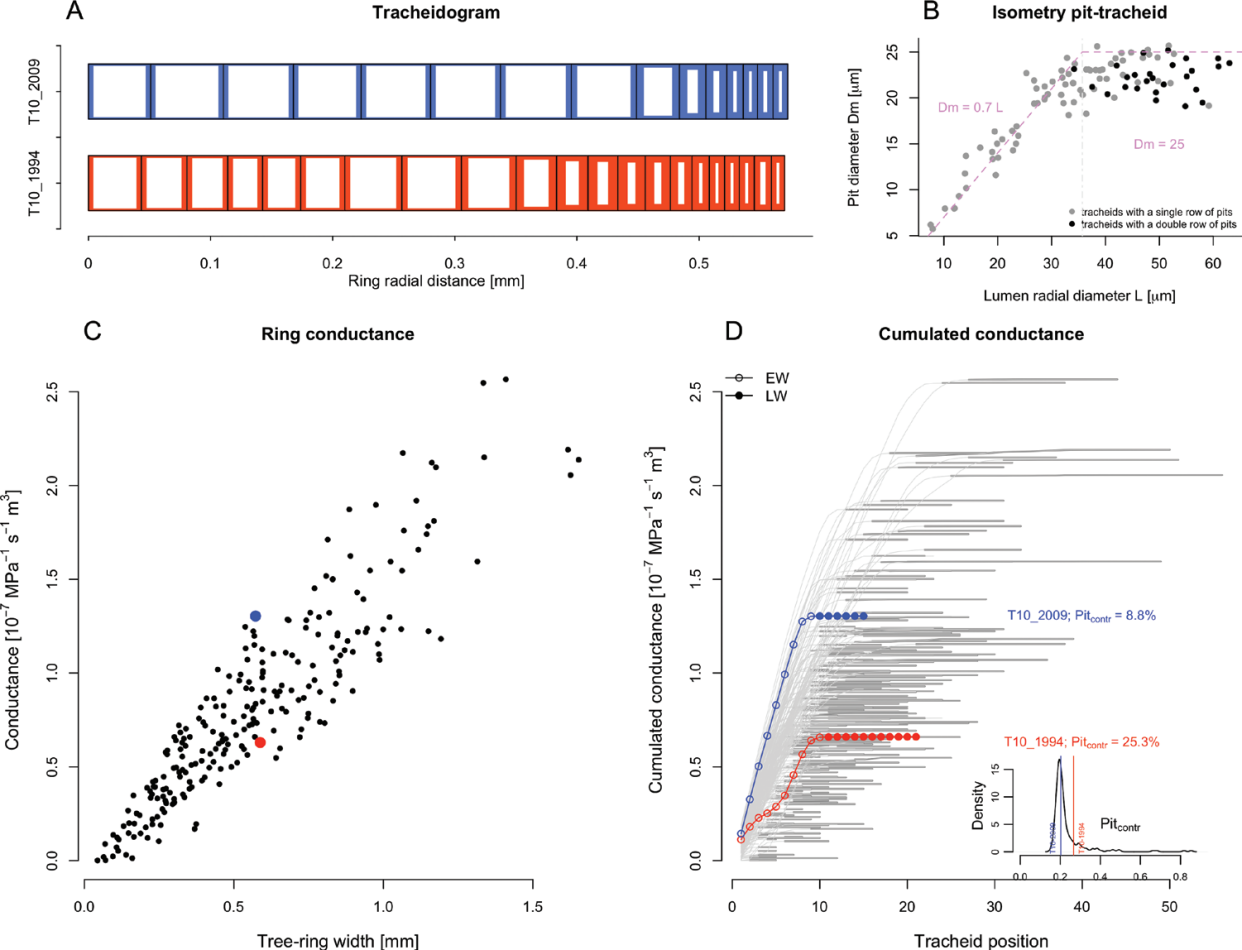
Application of the model to the anatomical dataset collected from eight *Larix sibirica* trees in southern Siberia. The dataset includes 240 tracheidograms from annual rings spanning the period from 1986 to 2015. (A, B) Results from the anatomical measurements. (A) An example of two selected tracheidograms from a tree (T10) with similar ring widths but different structures. See Table 3 for the measurements used to contstruct the tracheidograms. (B) An overview of pit size (*D*_m_) versus tracheid lumen diameter measurements (*L*). The isometric relationships applied are indicated by the violet lines and the labels. Within-pit isometric relationships applied in the model are indicated in Table 2. Model inputs are as follows: tracheid length *l* = 2.2 mm, tracheid tangential lumen diameter *TD* = 30 μm, maximum pit size max*D*_m_ = 25 μm, pit density α = 6.5×10^8^ m^–2^, mean thickness of margo strands *t*_f_ = 140 nm, and pith canal length *t*_a_ = tracheid wall thickness. The wall thickness *WT* corresponds to the measured data. (C, D) Tree-ring conductance changes as a function of tree-ring width (C) and as a cumulation of the tracheid contribution for each tracheidogram (D). Each point (in C) and line (in D) indicates a single annual ring/tracheidogram; thick grey lines in D indicate latewood cells. The colored points and lines refer to the two selected tracheidograms with similar tree-ring widths shown in A (see Table 3 for the calculated data). Open circles in D denote earlywood tracheids and closed circles denote latewood tracheids. The inset plot in D shows the frequency distribution of *Pit*_contr_ for all rings (*n*=240).

When the model was upscaled to the representative annual tracheidograms of eight mature *L. sibirica* trees covering a 30-year period (from 1986 to 2015), we observed a large variation in total ring conductance (Fig. 3C). The ring conductance of the analyzed tree rings showed a variation by three orders of magnitude, from a minimum of 1.17×10^–10^ MPa^–1^ s^–1^ m^3^ to a maximum of 2.57×10^–7^ MPa^–1^ s^–1^ m^3^. This variability is caused mainly by differences in ring width and cell structure, but also by radial files composed of differently sized tracheids (*L* and *WT*), which contribute differently to the total tree-ring conductance (Fig. 3D). For example, two annual rings of similar widths (~0.6 mm) but composed of differently sized tracheids (see schematic tracheidograms in Fig. 3A) differed in conductance by a factor of two (6.60×10^–8^ MPa^–1^ s^–1^ m^3^ versus 13.04×10^–8^ MPa^–1^ s^–1^ m^3^). The contribution of latewood tracheids to the conductivity of both rings was less than 1% (Table 3). Since the earlywood tracheids of the blue tracheidogram shown in Fig. 3A are considerably larger than those of the red tracheidogram, the pit resistance contributed to only 20.3% of the total tree-ring resistance (versus 26.7% for the red tracheidogram).

**Table 3.** Hydraulic measurements of the tracheidogram in Fig. 3

Location		Tracheid characteristics				Pit characteristics			Hydraulic resistances			Hydraulic conductances	
POS	EW/LW	*L* (µm)	*WT* (µm)	CWA (µm^2^)	*N*_pit_	*D*_m_ (µm)	*D*_a_ (µm)	*D*_t_ (µm)	*R*_wall_ (MPa s mm^–3^)	*R*_lum_ (MPa s mm^–3^)	*R* (MPa s mm^–3^)	*K* (kPa^–1^ s^–1^ mm^3^)	Pro.cum.Kh (%)
**T10 – 1994**													
**1**	EW	36.09	**3.58**	**472.91**	52	25.00	6.25	12.50	16	73	89	11.20	0.17
**2**	EW	29.68	**3.86**	**461.07**	42	20.77	5.19	10.39	39	107	146	6.84	0.27
**3**	EW	25.79	**3.81**	**425.10**	37	18.06	4.51	9.03	74	136	210	4.76	0.35
**4**	EW	20.76	**3.85**	**391.29**	30	14.53	3.63	7.27	204	210	415	2.41	0.38
**5**	EW	23.46	**3.91**	**417.59**	34	16.42	4.10	8.21	117	166	283	3.54	0.44
**6**	EW	28.21	**3.95**	**460.14**	40	19.75	4.94	9.87	50	119	169	5.92	0.53
**7**	EW	38.41	**4.12**	**563.87**	55	25.00	6.25	12.50	16	75	92	10.93	0.69
**8**	EW	40.12	**4.35**	**610.06**	57	25.00	6.25	12.50	16	75	91	11.02	0.86
**9**	EW	34.57	**5.12**	**661.61**	49	24.20	6.05	12.10	23	112	135	7.41	0.97
**10**	EW	20.94	**6.07**	**618.69**	30	14.66	3.66	7.33	263	325	589	1.70	1.00
**11**	LW	11.47	**7.14**	**592.18**	16	8.03	2.01	4.01	5 416	1 451	6 867	0.15	1.00
**12**	LW	9.49	**7.47**	**589.82**	14	6.64	1.66	3.32	14 194	2 442	16 636	0.06	1.00
**13**	LW	7.27	**7.56**	**563.84**	10	5.09	1.27	2.54	53 267	4 937	58 204	0.02	1.00
**14**	LW	6.72	**6.97**	**511.59**	10	4.71	1.18	2.35	73 167	5 564	78 731	0.01	1.00
**15**	LW	6.18	**5.75**	**415.97**	9	4.32	1.08	2.16	94 628	6 103	100 731	0.01	1.00
**16**	LW	3.60	**5.27**	**354.20**	5	2.52	0.63	1.26	1 297 171	33 072	1 330 243	0.00	1.00
**17**	LW	3.17	**4.69**	**311.06**	5	2.22	0.55	1.11	2 221 219	49 284	2 270 504	0.00	1.00
**18**	LW	3.26	**4.39**	**292.08**	5	2.28	0.57	1.14	1 821 187	43 883	1 865 069	0.00	1.00
**19**	LW	3.28	**4.47**	**297.50**	5	2.30	0.57	1.15	1 786 667	43 052	1 829 719	0.00	1.00
**20**	LW	5.33	**4.37**	**309.02**	8	3.73	0.93	1.87	157 023	8 500	165 523	0.01	1.00
**21**	LW	3.29	**3.75**	**249.41**	5	2.30	0.58	1.15	1 546 563	41 385	1 587 949	0.00	1.00
**T10 – 2009**													
**1**	EW	43.71	**3.65**	**537.91**	63	25.00	6.25	12.50	13	56	70	14.36	0.11
**2**	EW	52.40	**3.57**	**588.10**	75	25.00	6.25	12.50	11	44	55	18.27	0.25
**3**	EW	50.27	**3.51**	**562.95**	72	25.00	6.25	12.50	11	45	57	17.63	0.39
**4**	EW	48.22	**3.63**	**568.39**	69	25.00	6.25	12.50	12	49	61	16.31	0.51
**5**	EW	49.29	**3.76**	**595.77**	70	25.00	6.25	12.50	12	49	61	16.30	0.64
**6**	EW	49.66	**3.78**	**601.81**	71	25.00	6.25	12.50	12	49	61	16.37	0.76
**7**	EW	50.07	**3.95**	**632.68**	72	25.00	6.25	12.50	12	51	63	15.90	0.88
**8**	EW	44.53	**4.45**	**663.30**	64	25.00	6.25	12.50	14	67	81	12.31	0.98
**9**	EW	24.09	**5.56**	**601.55**	34	16.86	4.22	8.43	128	224	351	2.85	1.00
**10**	LW	9.49	**6.08**	**480.34**	14	6.64	1.66	3.32	11.999	1 905	13 905	0.07	1.00
**11**	LW	5.59	**5.79**	**412.11**	8	3.91	0.98	1.96	156 003	8 299	164 303	0.01	1.00
**12**	LW	3.98	**4.69**	**318.83**	6	2.78	0.70	1.39	711 823	22 694	734 517	0.00	1.00
**13**	LW	3.16	**4.28**	**284.05**	5	2.21	0.55	1.10	2 113 775	49 044	2 162 819	0.00	1.00
**14**	LW	3.90	**4.43**	**300.44**	6	2.73	0.68	1.37	746 575	23 792	770 367	0.00	1.00
**15**	LW	3.53	**4.22**	**283.31**	5	2.47	0.62	1.24	1 191 075	33 179	1 224 254	0.00	1.00

Measurements were made of the annual rings for 1994 and 2009 in tree T10. POS, tracheid position in the radial file; EW, earlywood tracheid; LW, latewood tracheid; *L*, lumen radial diameter; WT, cell wall thickness along the radial axis; CWA, cell wall area; *N*_pit_, number of pits; *D*_m_, pit diameter; *D*_a_, diameter of pit aperture; *D*_t_, diameter of pit torus; *R*_wall_, sum of pit resistance; *R*_lum_, lumen resistance; *R*, tracheid resistance; *K*, full tracheid conductance; Pro.cum.Kh, proportion of cumulative conductance to total ring conductance. *L* and *WT* are the input variables.

## Discussion

### Comparing the model results with those of the literature review

Upscaling wood hydraulic properties to a piece of wood by the integration of each hydraulic conduit and component is an immense challenge due to the large number and variability of morphological parameters involved. This is the reason why, despite the intimate relationship between xylem structure and function (e.g. Choat *et al.*, 2008; Pittermann *et al.*, 2010; Bouche *et al.*, 2014), relating xylem anatomy to its hydraulic properties relies on the average morphological characteristics of the species (e.g. Bouche *et al.*, 2014). In this work, we face the challenge of studying plant responses to environmental change at the cellular level by assuming the existence of isometric scaling among all components (lumen and wall) of the tracheid hydraulics. The proposed combined tracheid–pit model simply adds isometric relationships to existing models (Lancashire and Ennos, 2002; Pittermann *et al.*, 2006; Wilson *et al.*, 2008; Kedrov, 2012; Schulte *et al.*, 2015; Hacke *et al.* (2004), and anchors their applications to only those features for which measurements are facilitated by the recent improvements in efficiently assessing cell dimensions on wood cross-sections (e.g. von Arx and Carrer, 2014).

The results obtained—as applied to the case of Pinaceae—provide valuable information for quantifying the hydraulic plasticity among trees and over time. These data are key to assessing the contribution of each hydraulic component to the total flow resistance. Based on the assumption of the existence of isometric relationships within tracheid elements, we could, for example, assess whether pit structures do indeed play only a marginal role in obstructing the flow in large earlywood tracheids. Assessments of the contribution of total pit resistance (*Pit*_contr_) to the total ring resistance have ranged between 15.2% and 85.4% (mean 23.0%). These quantifications bring into question an early assessment of pit contribution, which estimated that pits are responsible for nearly two-thirds of the total tracheid resistance, regardless of tracheid size (Pittermann *et al.*, 2006). Our modeled values are lower than previous assessments because our calculation accounts for the variability in tracheid size (i.e. it is not based on an average tracheid/ring value). Our calculation accounts for the strong reduction in pit resistance in large tracheids, which are also particularly wide in the studied species. Indeed, the pit-induced resistances decrease at a faster rate than the lumen resistances with increasing tracheid size. This means that the water transport capability of the earlywood tissue is not predominantly limited by pits (Fig. 2). This contrasting result, however, might be biased by our different calculation of pit resistance. In addition, our model defines an upper pit size limit (up to 25 μm for our dominant *L. sibirica* trees), which is greater than the pit sizes (6–19 μm) used to build up the pit hydraulic model (Schulte, 2012; Schulte *et al.*, 2015).

It is also important to note that the transition from pit-dominated to lumen-dominated resistance occurs around the transition between earlywood and latewood, that is, at a tracheid radial diameter of ~18 μm. Thus, within the tree-ring tissue that is mostly devoted to water transport (but also more exposed to hydraulic failure and spreading; e.g. Pittermann and Sperry, 2003), the major role of pits is to provide safety against the spreading of cavitation (Hacke and Jansen, 2009). By contrast, the latewood pits play a completely different role. The contribution of latewood tracheids to ring conductance is particularly small, not only because of their narrow lumina but also because there are very few pits. It is therefore not surprising that the contribution of latewood tracheids to the total tree-ring conductance is limited to ~0.5%. However, the greatest number of pits on the tangential walls is usually observed in the latewood. Pits in these cells might therefore mainly provide radial water movement from xylem to cambium at the very beginning of the growing season when there is no transpiration (Koran, 1977; Kitin *et al.*, 2009).

These very different roles among tracheids of different sizes have a very large impact on model outcomes when these hydraulic quantifications are eventually performed at the tree-ring level, especially considering the significant inter-annual plasticity of wood anatomical structures (Fig. 3). Although the number of tracheids (and the ring width) is the main driver for such variability in the cumulated tree-ring hydraulic properties, even at comparable ring width, the ring hydraulic properties can differ by several fold due to the disproportionate effect of their structure on the hydraulic functioning, especially in wider rings. This also means that ring width—in contrast to earlywood width—is not always a reliable proxy for estimating the capacity of the tree ring to transport water.

### Future improvements

The effort toward a model-based assessment of hydraulic properties via anatomical measurements does not end with the presentation of this model. Although a comparison of our results with those of other studies indicates that our model provides plausible outputs at the level of a single pit or tracheid, there is still a large margin for verifications and improvements that need to be performed, especially with regard to quantifying the isometric relationships. More observational data linking the relationships among and between pit and tracheid structures need to be collected to consolidate and improve the isometric relationships at the species level. Another model assumption that requires further attention is related to the isometric relationships with and within the membrane elements. Indeed, available observational data have already shown that the pore size, even within the same membrane, can be highly variable (Domec *et al.*, 2006; Schulte *et al.*, 2015) and can have different contributions to the hydraulic properties (Li *et al.*, 2020). Finally, there is a need for research that focuses on validating model outputs with observational data. Facilitated by the use of advanced tools such as the Xyl’EM device (INRA France) and the Cavitron (Cochard *et al.*, 2013), these hydraulic measurements could be more systematically combined with anatomical measurements to overcome the limitations related to the use of average anatomical values for a species. By combining numerous measurements of hydraulic properties (Melcher *et al.*, 2012) with *in-situ* anatomical measurements (see e.g. Lens *et al.*, 2011; Fernandez *et al.*, 2019; Guérin *et al.*, 2020), it should be possible to provide a solid data basis with which our model approach can be cross-validated.

### Significance of the causal environment–structure–function–performance chain

Structure–function quantifications are essential for gathering valuable information about a plant’s capacity to allocate carbon and the way in which it allocates carbon into structure for ensuring proper functioning even during unfavorable periods. The previous year’s tree-ring structure is the legacy on which the current functioning depends. For example, a wood cell formed under certain environmental constraints needs to be able to fulfill the functional requirements to provide sufficient water supply to the photosynthetic and growing tissues (Tyree and Zimmermann, 2002; Holbrook, 2005; Brodribb and Cochard, 2009) under a variety of future environmental conditions (McDowell, 2011; Mayr *et al.*, 2020). The novel model proposed here considers a tree’s present performance as a function of conduit sizes resulting from structures formed under past environmental and climatic constraints.

Thanks to the assessment of isometric relationships among these anatomical structures (e.g. Williams *et al.*, 2019), it has been possible to merge existing models of flow rates through bordered pits (e.g. Lancashire and Ennos, 2002; Hacke *et al.*, 2004) and the tracheid lumen (Wilson *et al.*, 2008; Tanrattana *et al.*, 2019). This “morphological shortcut” allows us to upscale results to the local tissue level while still considering the specific environmental and/or ontogenetic signatures (i.e. the distance from the tree tip when performing comparisons among individuals) imprinted into the anatomical structures of the sap-conducting xylem. This framework creates the conditions to perform intraspecific investigations of structure–function relationships by accounting for specific structural characteristics at the location of the investigated tissue. Indeed, structure–function investigations performed so far have mainly compared hydraulic properties across species using a representative anatomical characterization of the species (e.g. Pittermann *et al.*, 2010; Bouche *et al.*, 2014, Anderegg *et al.*, 2016). Although this approach is essential for elucidating differences in drought vulnerability across species, it does not explain differences in responses among provenances, individual trees, or even along the life span of an individual, since the anatomical structure of the species is considered to be invariable. However, the variability in anatomical structure between and within individuals can be as relevant as the variability observed among species from the same family (Zobel and Buijtenen, 1989), inducing an important phenotypic variability in the hydraulic properties (González-Muñoz *et al*., 2018). Moreover, such tremendous variability could also be retrospectively linked to specific past environmental conditions (e.g. extreme events, experimental setups, climate changes, or environmental drift) to assess and interpret the mechanisms and causes beyond observed alterations in responses, resiliencies, and legacy effects (e.g. Dalla-Salda *et al.*, 2009; Anderegg *et al.*, 2013; Cailleret *et al.*, 2017; DeSoto *et al.*, 2020). This would make it possible to follow spatial and temporal patterns in hydraulic structural responses within the xylem. Annually dated time series of cell anatomical features, which can now also be translated into dated time series of hydraulic properties and carbon investment, can be exploited to investigate their relationship with environmental conditions and the subsequent tree growth performance (Fonti *et al.*, 2010; Fonti and Jansen, 2012).

## Conclusion

Several different approaches to assess xylem hydraulic properties (Melcher *et al.*, 2012) already exist, but the model proposed here is one that can be easily embraced by dendrochronologists and applied to investigate the hydraulic responses of conifers across time and environmental conditions. Specifically, the isometric scaling applied to existing consolidated partial models provides an opportunity to assess year-to-year and intra-annual variability of the functional properties of tracheids and removes the necessity of collecting data on pit sizes. This construct additionally allows the extension of current (and future) wood formation and structure models (e.g. the cambial module in the Vaganov–Shashkin model of tree-ring formation; see Vaganov *et al.*, 2011) to assess their functioning under different scenarios. Finally, the same input data can be easily used to assess mechanical properties and biomass investment. Such a range of functionalities provides the opportunity to investigate hydraulic–mechanical trade-offs and to assess construction or functional efficiencies per unit of biomass fixed into the wood structure. Thanks to these advantages, the model is suitable for studies at large spatial scales and at intra-annual resolution. However, more observational data are required to consolidate the isometric relationships and to fully validate the model. These improvements, integrated with other modules (e.g. to model wood structure and/or water flow) will allow advances to be made in our understanding of environmental impact on tree water transport.

## Data Availability

The model and the data supporting the findings of this study are available from the corresponding author, Patrick Fonti, upon request.
